# 6-(4-Meth­oxy­phen­yl)-7-phenyl-2,3-dihydro-1*H*-pyrrolizine-5-carbaldehyde

**DOI:** 10.1107/S1600536811033022

**Published:** 2011-08-27

**Authors:** Peter R. W. E. F. Keck, Dieter Schollmeyer, Stefan Laufer

**Affiliations:** a Eberhard-Karls-University Tübingen, Auf der Morgenstelle 8, 72076 Tübingen, Germany; b University Mainz, Institut of Organic Chemistry, Duesbergweg 10-14, 55099 Mainz, Germany

## Abstract

The 4-meth­oxy­phenyl residue in the title compound, C_21_H_19_NO_2_, is oriented at a dihedral angle of 54.6 (5)° with respect to the phenyl ring and at a dihedral angle of 52.5 (8)° with respect to the pyrrole ring of the pyrrolizine system. The phenyl ring is oriented at a dihedral angle of 36.2 (5)° with respect to the pyrrole ring. The meth­oxy group makes a C—C—O—C torsion angle of 3.8 (9)° with the attached benzene ring.

## Related literature

For the biological activity of aryl­pyrrolizines as mPGES-1 inhibitors, see: Liedtke *et al.* (2009 ▶). For dual COX/5-LOX inhibitors, see: Laufer (2001 ▶); Tries & Laufer (2001 ▶).
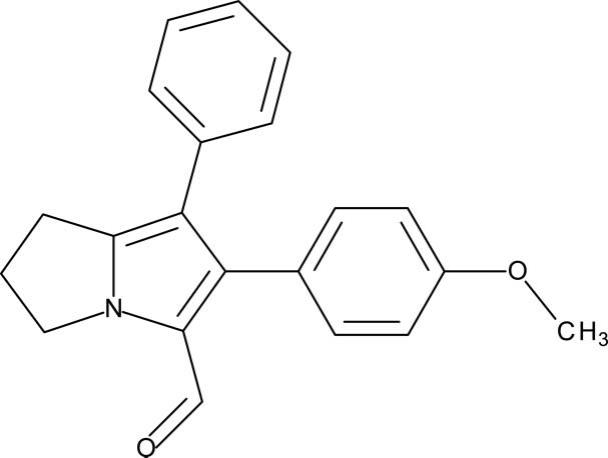


## Experimental

### 

#### Crystal data

C_21_H_19_NO_2_
*M* *_r_* = 317.37
Monoclinic, 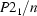

*a* = 12.2276 (17) Å
*b* = 9.1557 (10) Å
*c* = 15.462 (2) Å
β = 104.174 (11)°
*V* = 1678.3 (4) Å^3^
*Z* = 4
Mo *K*α radiation
μ = 0.08 mm^−1^
*T* = 193 K
0.30 × 0.20 × 0.07 mm

#### Data collection

Stoe IPDS 2T diffractometer
23718 measured reflections
4043 independent reflections
2868 reflections with *I* > 2σ(*I*)
*R*_int_ = 0.067

#### Refinement

*R*[*F* ^2^ > 2σ(*F* ^2^)] = 0.039
*wR*(*F* ^2^) = 0.102
*S* = 1.02
4043 reflections
218 parameters
H-atom parameters constrained
Δρ_max_ = 0.17 e Å^−3^
Δρ_min_ = −0.16 e Å^−3^

### 

Data collection: *X-AREA* (Stoe & Cie, 2010) ▶; cell refinement: *X-AREA* ▶; data reduction: *X-RED* (Stoe & Cie, 2010) ▶; program(s) used to solve structure: *SIR97* (Altomare *et al.*, 1999 ▶); program(s) used to refine structure: *SHELXL97* (Sheldrick, 2008 ▶); molecular graphics: *PLATON* (Spek, 2009 ▶); software used to prepare material for publication: *PLATON*.

## Supplementary Material

Crystal structure: contains datablock(s) I, global. DOI: 10.1107/S1600536811033022/si2370sup1.cif

Structure factors: contains datablock(s) I. DOI: 10.1107/S1600536811033022/si2370Isup2.hkl

Supplementary material file. DOI: 10.1107/S1600536811033022/si2370Isup3.cml

Additional supplementary materials:  crystallographic information; 3D view; checkCIF report

## Figures and Tables

**Fig. 1.**
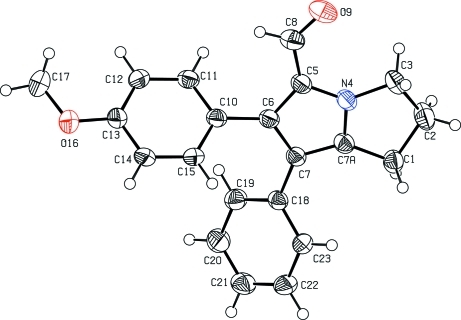
View of compound (I). Displacement ellipsoids are drawn at the 50% probability level.

## supplementary crystallographic information

### Comment

Based on ML3000 (Tries & Laufer, 2001 and Laufer, 2001) as dual COX/5-LOX inhibitor, we synthesized and evaluated inhibitors for the microsomal prostaglandin E_2_ synthase-1 (mPGES-1) (Liedtke *et al.*, 2009). The title compound was synthesized to obtain a template with a reactive group in position 5 of the pyrrolizine moiety which lead to series of differend derivates of the arylpyrrolizine scaffold.

Towards the unsaturated and planar part of the pyrrolizine residue the 4-methoxyphenyl residue is oriented at a dihedral angle of 52.5 (8)° and the plain phenyl ring is oriented at a dihedral angle of 36.2 (5)°. The two phenyl rings are oriented at a dihedral angle of 54.6 (5)° and both centromers show a distance of 4.89 (7) Å. The distance between the *para* C atoms of the rings (C13, C21) is 6.55 (9) Å. The methoxy group shows a torsion angle of 3.8 (9)° towards the phenyl ring.

### Experimental

The compound was prepared by Vilsmeyer reaction. Phosphoryl chloride (0.484 ml, 5.31 mmol) is added dropwise to ice-cooled solution of 1.18 ml dimethylformamide and 6-(4-methoxyphenyl)-7-phenyl-2,3-dihydro-1*H*-pyrrolizine (1.5 g, 5.11 mmol); the temperature is kept under 293 K during the addition. Then the mixture is stirred for 1 h at room temperature. Finally the mixture is heated to 333 K for 1 h. The mixture was cooled to 273 K, quenched by water and adjusted to pH 6 with aqueous sodium hydroxide solution 10%.

The product was collected as precipitated solid by filtration, was dissolved in dichloromethane and washed with water three times and finally dried over anhydrous sodium sulfate. The product was concentrated under vacuum. The residue was purified by column chromatography (SiO_2_, n-hexane / ethyl acetate: 2 + 1). Crystals of the title compound were obtained by slow evaporation of ethanol at room temperature.

### Refinement

Hydrogen atoms attached to carbons were placed at calculated positions with C—H = 0.95 Å (aromatic) or 0.99–1.00 Å (*sp*^3^ C-atom). All H atoms were refined with isotropic displacement parameters (set at 1.2–1.5 times of the *U*_eq_ of the parent atom).

### Crystal data

**Table:**

C_21_H_19_NO_2_	*F*(000) = 672
*M**_r_* = 317.37	*D*_x_ = 1.256 Mg m^−^^3^
Monoclinic, *P*2_1_/*n*	Mo *K*α radiation, λ = 0.71073 Å
Hall symbol: -P 2yn	Cell parameters from 18580 reflections
*a* = 12.2276 (17) Å	θ = 2.4–30°
*b* = 9.1557 (10) Å	µ = 0.08 mm^−^^1^
*c* = 15.462 (2) Å	*T* = 193 K
β = 104.174 (11)°	Plate, light brown
*V* = 1678.3 (4) Å^3^	0.30 × 0.20 × 0.07 mm
*Z* = 4	

### Data collection

**Table:**

Stoe IPDS 2T diffractometer	2868 reflections with *I* > 2σ(*I*)
Radiation source: sealed X-ray tube, 12 x 0.4 mm long-fine focus	*R*_int_ = 0.067
Detector resolution: 6.67 pixels mm^-1^	θ_max_ = 28.0°, θ_min_ = 2.4°
rotation method scans	*h* = −16→15
23718 measured reflections	*k* = −12→12
4043 independent reflections	*l* = −20→20

### Refinement

**Table:**

Refinement on *F*^2^	Primary atom site location: structure-invariant direct methods
Least-squares matrix: full	Secondary atom site location: difference Fourier map
*R*[*F*^2^ > 2σ(*F*^2^)] = 0.039	Hydrogen site location: inferred from neighbouring sites
*wR*(*F*^2^) = 0.102	H-atom parameters constrained
*S* = 1.02	*w* = 1/[σ^2^(*F*_o_^2^) + (0.047*P*)^2^ + 0.1927*P*] where *P* = (*F*_o_^2^ + 2*F*_c_^2^)/3
4043 reflections	(Δ/σ)_max_ < 0.001
218 parameters	Δρ_max_ = 0.17 e Å^−^^3^
0 restraints	Δρ_min_ = −0.16 e Å^−^^3^

### Special details

**Table:**

Geometry. All e.s.d.'s (except the e.s.d. in the dihedral angle between two l.s. planes) are estimated using the full covariance matrix. The cell e.s.d.'s are taken into account individually in the estimation of e.s.d.'s in distances, angles and torsion angles; correlations between e.s.d.'s in cell parameters are only used when they are defined by crystal symmetry. An approximate (isotropic) treatment of cell e.s.d.'s is used for estimating e.s.d.'s involving l.s. planes.
Refinement. Refinement of *F*^2^ against ALL reflections. The weighted *R*-factor *wR* and goodness of fit *S* are based on *F*^2^, conventional *R*-factors *R* are based on *F*, with *F* set to zero for negative *F*^2^. The threshold expression of *F*^2^ > σ(*F*^2^) is used only for calculating *R*-factors(gt) *etc*. and is not relevant to the choice of reflections for refinement. *R*-factors based on *F*^2^ are statistically about twice as large as those based on *F*, and *R*- factors based on ALL data will be even larger.

### Fractional atomic coordinates and isotropic or equivalent isotropic displacement parameters (Å^2^)

**Table:**

	*x*	*y*	*z*	*U*_iso_*/*U*_eq_	
C1	0.06152 (12)	0.14978 (15)	0.66247 (9)	0.0393 (3)	
H1A	0.0946	0.2238	0.7081	0.047*	
H1B	0.1108	0.0625	0.6709	0.047*	
C2	−0.05958 (14)	0.10999 (18)	0.66633 (10)	0.0517 (4)	
H2A	−0.0599	0.0171	0.6991	0.062*	
H2B	−0.0917	0.1877	0.6972	0.062*	
C3	−0.12865 (12)	0.09433 (16)	0.56986 (10)	0.0432 (3)	
H3A	−0.1354	−0.0092	0.5508	0.052*	
H3B	−0.2050	0.1368	0.5617	0.052*	
N4	−0.06033 (9)	0.17804 (11)	0.52170 (7)	0.0359 (2)	
C5	−0.07535 (10)	0.22928 (14)	0.43583 (9)	0.0352 (3)	
C6	0.02545 (10)	0.29896 (13)	0.43203 (8)	0.0319 (3)	
C7	0.10129 (10)	0.28749 (13)	0.51738 (8)	0.0319 (3)	
C7A	0.04412 (10)	0.21009 (13)	0.57039 (8)	0.0339 (3)	
C8	−0.17589 (11)	0.20663 (15)	0.36689 (10)	0.0430 (3)	
H8	−0.1776	0.2450	0.3095	0.052*	
O9	−0.25971 (9)	0.14180 (13)	0.37601 (8)	0.0582 (3)	
C10	0.05027 (10)	0.36298 (13)	0.35126 (8)	0.0321 (3)	
C11	−0.01913 (10)	0.46703 (13)	0.29946 (8)	0.0343 (3)	
H11	−0.0849	0.4985	0.3165	0.041*	
C12	0.00522 (10)	0.52607 (14)	0.22357 (8)	0.0358 (3)	
H12	−0.0439	0.5962	0.1889	0.043*	
C13	0.10122 (11)	0.48215 (15)	0.19885 (8)	0.0370 (3)	
C14	0.17152 (11)	0.37719 (16)	0.24926 (9)	0.0407 (3)	
H14	0.2371	0.3459	0.2320	0.049*	
C15	0.14613 (10)	0.31872 (15)	0.32406 (9)	0.0369 (3)	
H15	0.1946	0.2470	0.3578	0.044*	
O16	0.13358 (9)	0.53308 (12)	0.12557 (7)	0.0498 (3)	
C17	0.06605 (15)	0.64537 (19)	0.07522 (11)	0.0580 (4)	
H17A	−0.0108	0.6088	0.0513	0.087*	
H17B	0.0985	0.6748	0.0259	0.087*	
H17C	0.0640	0.7297	0.1138	0.087*	
C18	0.21576 (10)	0.34911 (13)	0.54706 (8)	0.0315 (3)	
C19	0.24241 (11)	0.48377 (14)	0.51540 (9)	0.0397 (3)	
H19	0.1856	0.5375	0.4749	0.048*	
C20	0.35037 (12)	0.54021 (16)	0.54212 (10)	0.0462 (3)	
H20	0.3675	0.6313	0.5192	0.055*	
C21	0.43355 (12)	0.46435 (16)	0.60222 (9)	0.0458 (3)	
H21	0.5078	0.5029	0.6203	0.055*	
C22	0.40810 (11)	0.33296 (16)	0.63563 (9)	0.0428 (3)	
H22	0.4646	0.2817	0.6778	0.051*	
C23	0.30042 (11)	0.27471 (14)	0.60821 (8)	0.0364 (3)	
H23	0.2841	0.1833	0.6313	0.044*	

### Atomic displacement parameters (Å^2^)

**Table:**

	*U*^11^	*U*^22^	*U*^33^	*U*^12^	*U*^13^	*U*^23^
C1	0.0432 (7)	0.0409 (7)	0.0367 (7)	0.0017 (5)	0.0154 (5)	0.0031 (5)
C2	0.0539 (9)	0.0616 (9)	0.0454 (8)	−0.0123 (7)	0.0234 (7)	0.0019 (7)
C3	0.0396 (7)	0.0450 (7)	0.0501 (8)	−0.0072 (6)	0.0209 (6)	0.0019 (6)
N4	0.0306 (5)	0.0407 (5)	0.0386 (6)	−0.0036 (4)	0.0129 (4)	0.0008 (5)
C5	0.0296 (6)	0.0411 (6)	0.0361 (6)	−0.0010 (5)	0.0102 (5)	−0.0007 (5)
C6	0.0268 (6)	0.0368 (6)	0.0324 (6)	0.0011 (4)	0.0080 (5)	−0.0010 (5)
C7	0.0289 (6)	0.0363 (6)	0.0314 (6)	0.0011 (5)	0.0094 (5)	−0.0005 (5)
C7A	0.0323 (6)	0.0369 (6)	0.0342 (6)	0.0004 (5)	0.0116 (5)	−0.0018 (5)
C8	0.0310 (7)	0.0497 (7)	0.0469 (8)	−0.0026 (6)	0.0070 (6)	−0.0037 (6)
O9	0.0336 (6)	0.0685 (7)	0.0695 (7)	−0.0141 (5)	0.0069 (5)	−0.0018 (6)
C10	0.0260 (6)	0.0391 (6)	0.0304 (6)	−0.0005 (5)	0.0055 (4)	−0.0013 (5)
C11	0.0271 (6)	0.0411 (6)	0.0349 (6)	0.0036 (5)	0.0079 (5)	−0.0021 (5)
C12	0.0323 (6)	0.0390 (6)	0.0343 (6)	0.0043 (5)	0.0046 (5)	0.0034 (5)
C13	0.0326 (6)	0.0461 (7)	0.0323 (6)	−0.0012 (5)	0.0082 (5)	0.0038 (5)
C14	0.0270 (6)	0.0565 (8)	0.0405 (7)	0.0073 (5)	0.0120 (5)	0.0070 (6)
C15	0.0267 (6)	0.0470 (7)	0.0362 (6)	0.0056 (5)	0.0059 (5)	0.0064 (5)
O16	0.0450 (6)	0.0659 (6)	0.0418 (5)	0.0064 (5)	0.0170 (4)	0.0167 (5)
C17	0.0609 (10)	0.0662 (10)	0.0462 (9)	0.0048 (8)	0.0120 (7)	0.0216 (8)
C18	0.0286 (6)	0.0388 (6)	0.0278 (6)	−0.0005 (5)	0.0085 (4)	−0.0034 (5)
C19	0.0360 (7)	0.0394 (6)	0.0413 (7)	−0.0013 (5)	0.0047 (5)	0.0023 (5)
C20	0.0433 (8)	0.0435 (7)	0.0496 (8)	−0.0106 (6)	0.0071 (6)	0.0017 (6)
C21	0.0346 (7)	0.0561 (8)	0.0433 (8)	−0.0096 (6)	0.0033 (6)	−0.0054 (6)
C22	0.0337 (7)	0.0550 (8)	0.0355 (7)	0.0004 (6)	0.0005 (5)	0.0007 (6)
C23	0.0339 (6)	0.0435 (7)	0.0307 (6)	−0.0004 (5)	0.0057 (5)	0.0025 (5)

### Geometric parameters (Å, °)

**Table:**

C1—C7A	1.4929 (18)	C11—H11	0.9500
C1—C2	1.540 (2)	C12—C13	1.3805 (18)
C1—H1A	0.9900	C12—H12	0.9500
C1—H1B	0.9900	C13—O16	1.3701 (16)
C2—C3	1.530 (2)	C13—C14	1.3935 (18)
C2—H2A	0.9900	C14—C15	1.3771 (19)
C2—H2B	0.9900	C14—H14	0.9500
C3—N4	1.4645 (16)	C15—H15	0.9500
C3—H3A	0.9900	O16—C17	1.4247 (18)
C3—H3B	0.9900	C17—H17A	0.9800
N4—C7A	1.3466 (16)	C17—H17B	0.9800
N4—C5	1.3773 (17)	C17—H17C	0.9800
C5—C6	1.4019 (17)	C18—C19	1.3943 (18)
C5—C8	1.4314 (18)	C18—C23	1.3965 (17)
C6—C7	1.4189 (17)	C19—C20	1.3833 (19)
C6—C10	1.4768 (17)	C19—H19	0.9500
C7—C7A	1.3941 (17)	C20—C21	1.385 (2)
C7—C18	1.4743 (17)	C20—H20	0.9500
C8—O9	1.2221 (17)	C21—C22	1.375 (2)
C8—H8	0.9500	C21—H21	0.9500
C10—C11	1.3915 (17)	C22—C23	1.3873 (18)
C10—C15	1.3987 (17)	C22—H22	0.9500
C11—C12	1.3889 (18)	C23—H23	0.9500
			
C7A—C1—C2	102.16 (11)	C12—C11—H11	119.1
C7A—C1—H1A	111.3	C10—C11—H11	119.1
C2—C1—H1A	111.3	C13—C12—C11	119.56 (11)
C7A—C1—H1B	111.3	C13—C12—H12	120.2
C2—C1—H1B	111.3	C11—C12—H12	120.2
H1A—C1—H1B	109.2	O16—C13—C12	124.55 (12)
C3—C2—C1	106.90 (11)	O16—C13—C14	115.71 (12)
C3—C2—H2A	110.3	C12—C13—C14	119.73 (12)
C1—C2—H2A	110.3	C15—C14—C13	120.19 (12)
C3—C2—H2B	110.3	C15—C14—H14	119.9
C1—C2—H2B	110.3	C13—C14—H14	119.9
H2A—C2—H2B	108.6	C14—C15—C10	121.18 (12)
N4—C3—C2	101.49 (11)	C14—C15—H15	119.4
N4—C3—H3A	111.5	C10—C15—H15	119.4
C2—C3—H3A	111.5	C13—O16—C17	116.83 (12)
N4—C3—H3B	111.5	O16—C17—H17A	109.5
C2—C3—H3B	111.5	O16—C17—H17B	109.5
H3A—C3—H3B	109.3	H17A—C17—H17B	109.5
C7A—N4—C5	110.43 (10)	O16—C17—H17C	109.5
C7A—N4—C3	114.25 (11)	H17A—C17—H17C	109.5
C5—N4—C3	135.27 (11)	H17B—C17—H17C	109.5
N4—C5—C6	106.52 (11)	C19—C18—C23	118.09 (11)
N4—C5—C8	123.78 (11)	C19—C18—C7	120.98 (11)
C6—C5—C8	129.66 (12)	C23—C18—C7	120.93 (11)
C5—C6—C7	107.96 (11)	C20—C19—C18	120.89 (12)
C5—C6—C10	125.52 (11)	C20—C19—H19	119.6
C7—C6—C10	126.37 (11)	C18—C19—H19	119.6
C7A—C7—C6	106.08 (11)	C19—C20—C21	120.23 (13)
C7A—C7—C18	125.87 (11)	C19—C20—H20	119.9
C6—C7—C18	128.00 (11)	C21—C20—H20	119.9
N4—C7A—C7	108.99 (11)	C22—C21—C20	119.64 (13)
N4—C7A—C1	110.30 (11)	C22—C21—H21	120.2
C7—C7A—C1	140.70 (12)	C20—C21—H21	120.2
O9—C8—C5	125.17 (14)	C21—C22—C23	120.45 (13)
O9—C8—H8	117.4	C21—C22—H22	119.8
C5—C8—H8	117.4	C23—C22—H22	119.8
C11—C10—C15	117.59 (11)	C22—C23—C18	120.67 (12)
C11—C10—C6	122.40 (11)	C22—C23—H23	119.7
C15—C10—C6	120.01 (11)	C18—C23—H23	119.7
C12—C11—C10	121.74 (12)		
			
C7A—C1—C2—C3	21.22 (15)	C5—C6—C10—C11	55.01 (18)
C1—C2—C3—N4	−21.05 (15)	C7—C6—C10—C11	−129.87 (14)
C2—C3—N4—C7A	13.49 (15)	C5—C6—C10—C15	−124.61 (14)
C2—C3—N4—C5	−169.27 (14)	C7—C6—C10—C15	50.51 (18)
C7A—N4—C5—C6	−0.94 (14)	C15—C10—C11—C12	−0.26 (18)
C3—N4—C5—C6	−178.26 (13)	C6—C10—C11—C12	−179.88 (11)
C7A—N4—C5—C8	177.15 (12)	C10—C11—C12—C13	−0.67 (19)
C3—N4—C5—C8	−0.2 (2)	C11—C12—C13—O16	−179.91 (12)
N4—C5—C6—C7	0.34 (14)	C11—C12—C13—C14	1.2 (2)
C8—C5—C6—C7	−177.60 (13)	O16—C13—C14—C15	−179.78 (13)
N4—C5—C6—C10	176.21 (11)	C12—C13—C14—C15	−0.8 (2)
C8—C5—C6—C10	−1.7 (2)	C13—C14—C15—C10	−0.2 (2)
C5—C6—C7—C7A	0.35 (14)	C11—C10—C15—C14	0.68 (19)
C10—C6—C7—C7A	−175.48 (11)	C6—C10—C15—C14	−179.68 (12)
C5—C6—C7—C18	−177.23 (12)	C12—C13—O16—C17	3.9 (2)
C10—C6—C7—C18	6.9 (2)	C14—C13—O16—C17	−177.17 (13)
C5—N4—C7A—C7	1.19 (14)	C7A—C7—C18—C19	−142.11 (13)
C3—N4—C7A—C7	179.11 (10)	C6—C7—C18—C19	35.02 (19)
C5—N4—C7A—C1	−177.99 (10)	C7A—C7—C18—C23	37.43 (18)
C3—N4—C7A—C1	−0.06 (15)	C6—C7—C18—C23	−145.44 (13)
C6—C7—C7A—N4	−0.93 (14)	C23—C18—C19—C20	1.60 (19)
C18—C7—C7A—N4	176.71 (11)	C7—C18—C19—C20	−178.85 (13)
C6—C7—C7A—C1	177.85 (15)	C18—C19—C20—C21	−1.1 (2)
C18—C7—C7A—C1	−4.5 (2)	C19—C20—C21—C22	−0.4 (2)
C2—C1—C7A—N4	−13.35 (14)	C20—C21—C22—C23	1.3 (2)
C2—C1—C7A—C7	167.88 (16)	C21—C22—C23—C18	−0.7 (2)
N4—C5—C8—O9	1.0 (2)	C19—C18—C23—C22	−0.70 (19)
C6—C5—C8—O9	178.61 (14)	C7—C18—C23—C22	179.74 (12)
